# STC2 Is a Potential Prognostic Biomarker for Pancreatic Cancer and Promotes Migration and Invasion by Inducing Epithelial–Mesenchymal Transition

**DOI:** 10.1155/2019/8042489

**Published:** 2019-07-15

**Authors:** Chen Lin, Lina Sun, Shenglei Huang, Xiangqun Weng, Zhixian Wu

**Affiliations:** ^1^Department of General Surgery, Fuzhou General Hospital (Dongfang Hospital), Xiamen University, Fuzhou, Fujian 350025, China; ^2^Department of Presbyatrics, Fuzhou General Hospital (Dongfang Hospital), Xiamen University, Fuzhou, Fujian 350025, China; ^3^Department of Hepatobiliary Disease, Fuzhou General Hospital (Dongfang Hospital), Xiamen University, Fuzhou, Fujian 350025, China; ^4^Department of Hepatobiliary Disease, The 900th Hospital of the People's Liberation Army Joint Service Support Force, Fuzhou, Fujian 350025, China; ^5^Department of General Practice, Fuzhou General Hospital (Dongfang Hospital), Xiamen University, Fuzhou, Fujian 350025, China

## Abstract

Aberrant expression of stanniocalcin 2 (STC2) is implicated in cancer development. STC2 acts as a tumor promoter to drive some cancers. However, its contribution to the development of pancreatic cancer remains unclear. This study showed that the expression of STC2 was significantly upregulated in pancreatic cancer tissues. Moreover, its expression was positively correlated with tumor size and lymph node metastasis and negatively correlated with 5-year survival rate of pancreatic cancer patients. Additionally, the expression levels of STC2 were a novel biomarker for predicting overall survival rate after surgery. Furthermore, overexpression of STC2 could promote the proliferation, migration, and invasion of pancreatic cancer cell lines, while knocking down of STC2 led to antiproliferation and antimetastasis activities. Further mechanistic investigations revealed that the expression of STC2 could significantly promote the epithelial–mesenchymal transition (EMT) in pancreatic cancer cells. These data indicated that the overexpression of STC2 in pancreatic cancer contributes to the metastasis through the promotion of EMT, suggesting that STC2 is a potential prognostic biomarker and therapeutic target for pancreatic cancer.

## 1. Introduction

Pancreatic carcinoma is a common digestive system cancer and the fourth commonest of cancer-related deaths among both genders in the United States [1]. Remarkably, the cancer statistics of 2018 showed that the estimated new cases of pancreatic cancer have been the eighth highest incidence rate among women, and slipped out of the top ten among men, suggesting that the medical therapy for pancreatic carcinoma is inefficient. Due to lack of early diagnosis, the pancreatic cancer patients are inoperable, and chemotherapy is the only treatment option for many patients. Pancreatic cancer shows no significant symptoms in the early stage, and the evident symptoms often occurred in the late stage, resulting in unsatisfactory curative results, so early diagnosis and effective treatment could improve overall survival and disease prognosis. Importantly, the drug resistance of chemotherapy is an important limiting factor for the therapy of pancreatic carcinoma, many factors contribute to the poor sensitivity of pancreatic cancer cells to chemotherapy, and compensatory pathways have been reported as activated statue in the drug metabolism [2]. Thus, novel therapeutic targets need to be urgently developed for the treatment of pancreatic cancer.

Stanniocalcin 2 (STC2) is a human ortholog of fish stanniocalcin (STC) that is widely expressed in various organs and tissues [3, 4]. STC2 may play a role in glucose homeostasis and phosphorus metastasis [5]. Its significance has been noted in tumor development and progression. STC2 was implicated in breast, cervical, and ovarian cancers [6–9], suggesting that it has hormone-specific or -dependent action in these malignancies. Moreover, it was reported to be involved in digestive system tumors, including esophageal, gastric, colon, and liver cancers [10–13], and respiratory system cancers including laryngeal and lung cancers [14, 15]. It also has effects on renal carcinoma and prostate cancer [16, 17]. Another notable study about STC2 was its significance in prognostic prediction. Esseghir S. and coauthors identified that STC2 was a prognostic marker in breast cancer [18]. And Wang J. et al. evaluated that the secreted STC2 in peripheral blood may be a potential biomarker for the screening diagnosis and prognosis of gastric cancer [19]. Further study also revealed that the overexpression of STC2 predicted poor prognosis in nasopharyngeal carcinoma [20], laryngeal squamous carcinoma [21], and hepatocellular carcinoma [22]. And STC2 could significantly activate the mitogen-activated protein kinase (MAPK) and phosphatidylinositol 3-kinase/protein kinase B (PI3K/AKT) signaling pathways in some cancers [11, 23]. Conversely, some reports showed that STC2 was a tumor suppressor. In some breast cancer cells, STC2 inhibited migration and invasion via inhibition of protein kinase C (PKC) signaling [24]. Therefore, the function of STC2 in the development of some cancers remains controversial. Notably, STC2 was reported to mediate drug resistance [9, 25, 26], which was a key factor in cancer treatment.

However, the significance of STC2 expression has not been reported in pancreatic cancer. Importantly, the underlying regulatory mechanisms in the proliferation and metastasis have not been explored. Therefore, in this study, we confirmed the significance of STC2 expression in pancreatic carcinoma. The aberrant expression was significantly correlated with the development of pancreatic carcinoma, indicating that STC2 was a potential biomarker for diagnosis of pancreatic cancer. This is the first study to show that elevated expression of STC2 acted as a tumor promoter in pancreatic cancer cell. Our data demonstrated that STC2 was a potential prognostic biomarker and therapeutic target for pancreatic cancer.

## 2. Materials and Methods

### 2.1. Reagents

Dulbecco's Modified Eagle's Medium (DMEM) (Cat. 11965084), RMPI1640 (Cat. 11875093), Ham's F12 (Cat. 11320033), Leibovitz's L-15 medium (Cat. 41300070), fetal bovine serum (FBS) (Cat. 10500064), insulin (Cat. A11382IJ), and penicillin-streptomycin (Cat. 15140148) were purchased from Gibco (MA, USA); RNA extraction reagent (Cat. 10606ES60), first strand cDNA synthesis mixture (Cat. 11137ES70), qPCR SYBR Green kit (Cat. 11203ES03), lentivirus concentration solution kit (Cat. 41101ES50), enhanced ECL chemiluminescent substrate kit (Cat. 36222ES76), transferrin (Cat.40102ES60), and cell cycle analysis kit (Cat. 40301ES50) were obtained from Yeasen (Shanghai, China); anti-GAPDH (5174S), anti-Snail (3879S), anti-Vimentin (5741S), anti-E-cadherin (3195S), anti-twist1 (46702S), and anti-*β*-Catenin (8480S) were purchased from Cell Signaling Technology (MA, USA); recombinant human EGF protein (P01133) was purchased from R&D System; HRP-conjugated goat anti-mouse IgG (Cat. SA00001-1), HRP-conjugated goat anti-rabbit IgG (Cat. SA00001-2), and anti-STC2 antibody (Cat. 60063-1-Ig) were from ProteinTech (Chicago, USA); hydrocortisone (Cat. S1696) was purchased from Selleck (Houston, USA); IHC kit (Cat. KIT-5020) was obtained from MXB Biotechnology (Fuzhou, China); PrimeSTAR HS DNA Polymerase (Cat. R045B) was from Takara Bio (Shiga, Japan); dual luciferase reporter kit (Cat. RG028) was obtained from Beyotime Bio (Shanghai, China); Matrigel matrix (Cat. 356234) was from BD Biosciences (San Jose, CA, USA)

### 2.2. Clinical Samples

The clinical tumor and corresponding normal specimens were collected from 98 cases of pancreatic cancer patients, who were operated at the Fuzhou General Hospital (Dongfang Hospital) of Xiamen University. All patients who did not undergo prior therapy were enrolled in this study, which was approved by the Ethics Commission of Xiamen University. All patients provided written informed consent. The tissue collection was conducted in the surgical operation and immediately stored at ultralow temperature freezer (-80°C).

### 2.3. RNA Extraction and Quantitative Reverse-Transcription-PCR (qRT-PCR) Analysis

Total cellular RNA was extracted by TRIzol reagent (Cat. 15596026, Invitrogen). The first strand was synthesized with first strand cDNA synthesis kit (Cat. 11119ES60, Yeasen). qRT-PCR was used to analyze the expression of STC2 in the samples. Target gene expression levels were normalized based on *β*-actin expression level. The primers for qRT-PCR were as follows: STC2 (forward, 5′- TCT TGT GAG ATT CGG GGC TT- 3′; reverse, 5′- ACA GGT CGT GCT TGA GGT AG- 3′); *β*-actin (forward, 5′-CAT CCG CAA AGA CCT GTA CG-3′; reverse, 5′-CCT GCT TGC TGA TCC ACA TC-3′). All primers were synthesized by BGI Tech.

### 2.4. Immunohistochemistry (IHC)

Immunohistochemistry (IHC) was used to test the expression of STC2 in pancreatic cancer tissues according to the IHC protocol. In detail, the slices were firstly subjected into deparaffinization with ethanol. And the antigen unmasking was conducted with 10 mM sodium citrate buffer (pH 6.0) at a boiling temperature for 10 min. Also, the slices were incubated with 3% hydrogen peroxide for 10 min at room temperature. Subsequently, the goat serum was used as blocking solution. Then the specimens were probed with anti-STC2 antibody (1:100) and, after, incubated with secondary antibody; the slices were stained with DAB reagent. The result was confirmed by the department of pathology.

### 2.5. Plasmid Construction and Transfection

The full-length coding sequence of STC2 was cloned into pLV-IRES-eGFP vector, and the shRNA of STC2 was inserted into pLKO.1 vector. The shRNA sequence of STC2 was 5′- AGG GCA AGT CAT TCA TCA AAG C -3′. The constructs were confirmed by Sanger sequencing in Invitrogen. And the packaging and infection of overexpression and knock-down vectors were briefly described as follows: the overexpressing pLV-IRES-eGFP-STC2 or knocking down plasmids pLKO.1-STC2 were, respectively, cotransfected with pCMV-VSV-G and pCMV-deltaR8.91 plasmids into HEK293T cells with lipofectamine 2000 and cultured for 48 h. The supernatant was collected and filtrated through 0.45 *μ*m filter membrane to remove the cells and fragments. Then the collected solution was subjected to lentivirus concentration solution kit according to the manufacturer's instruction. The enriched lentivirus solution was stored in ultralow temperature freezer.

### 2.6. Cell Culture

All cell lines in this project were obtained from the American Type Culture Collection (ATCC). HEK293T (ACS-4500), PANC-1 (CRL-1469), and Capan-1(HTB79) cells were cultured in DMEM, supplemented with 10% FBS and 100 U/mL penicillin, and 100 *μ*g/mL streptomycin in humidified 5% CO2 incubator at 37°C. HPAC cells were cultured in 1:1 ratio of DMEM and Ham's F12 medium supplemented with 0.002 mg/ml insulin, 0.005 mg/ml transferrin, 40 ng/ml hydrocortisone, 10 ng/ml epidermal growth factor, and 5% FBS. The BxPC-3 cells were obtained in RPMI-1640 medium with the 10% FBS, and the SW 1990 cells were cultured in L-15 medium with 10% FBS supplement. All cells were cultured in 25 cm^2^ flask, and the cells were subjected to passage when the confluence of cell reached about 90%. The cells were digested with 0.25% EDTA trypsin for 3 to 5 min, and the complete medium was added to terminate the digestion. And a subcultivation ration was about 1:4, and, after about 2 days, fresh complete medium should be exchanged.

### 2.7. Western Blotting Assay

The cells were collected with cell scraper, and the cells were washed with cold PBS twice. The cells were lysed with RIPA lysis supplied with protease inhibitor on ice for 30 min. And the cell lysis was centrifuged at 12000 g at 4°C. And the supernatant was collected and supplied with 5% loading buffer to heater at 99°C for 10 min. The cell lysates were electrophoresed on 10% SDS-PAGE and transferred onto polyvinylidene difluoride (PVDF) membranes. The membranes were blocked with 5% milk in TBST (10 mM Tris-HCl, pH 8.0, 150 mM NaCl, and 0.1% Tween 20) for 1 h and incubated with primary antibody in 5% BSA in TBST overnight at 4°C. The membranes were washed three times with TBST and then incubated for 1 h at room temperature in TBST containing horseradish peroxidase-linked anti-mouse or rabbit IgG. After washing with TBST three times, the immunoreactive bands were visualized using an enhanced ECL chemiluminescent substrate kit (Yeasen)

### 2.8. Cell Count Kit-8 (CCK-8) Assay

The cells were seeded in 96-well plates, at a density of 5000 cells per well, and then cultured for 18 h. The cells were treated with overexpression or knocking down lentivirus solutions for indicated times, and the CCK-8 reagent was added into the cells for a further 3 h culture, and the absorbance was valued at 450 nm on the microplate reader.

### 2.9. Transwell Assay

The invasion activity was evaluated by Transwell assay using 6.5 mm Transwell with 8.0 *μ*m pore polycarbonate membrane cell culture inserts. The inserts were pretreated with Matrigel matrix (BD Biosciences), and seeded cells were cultured for further 24 h culture. Then the invasive cells below the membrane were fixed with 4% paraformaldehyde and subjected to crystal violet staining. The results were recorded with a microscope, and the number of invasive cells was counted from three random fields.

### 2.10. Wound Healing Assay

The cells were seeded into 6-well plate and further cultured for 24 h. Then, the cells were scratched with sterile pipette tips and washed with phosphate buffer saline (PBS) three times and then subjected to indicated treatments for further 24 h. The images were recorded with a microscope.

### 2.11. TCGA Database Analysis

A total of 178 cases of pancreatic cancer patient samples from the Cancer Genome Atlas (TCGA) database were used to evaluate the correlation between STC2 and E-cadherin or Vimentin. The correlation module represents the expression scatterplots between STC2 and indicated genes in pancreatic cancer. The Spearman's correlation and estimated statistical significance were used to analyze the results.

### 2.12. Statistical Analysis

The results were obtained from at least three independent experiments. All the data were expressed as mean ± standard deviation (SD). The statistical significances of differences were analyzed using an analysis of variance or Student test.* p*<0.05 was considered as significant difference.

## 3. Results

### 3.1. The Overexpression of STC2 Predicts a Poor Prognosis in Pancreatic Cancer

**Figure 1:**
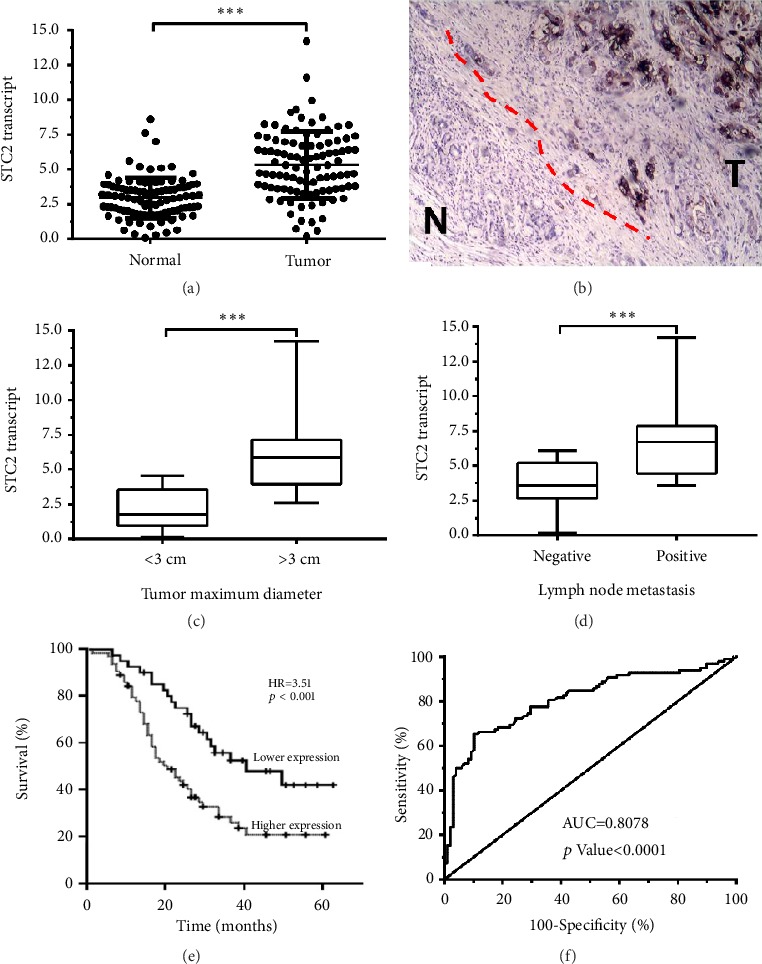
STC2 was overexpressed in pancreatic cancer tissues and correlated with the development and progression of cancer. (a) qRT-PCR was subjected to analyze the different mRNA expression of STC2 in the 98 cases of pancreatic cancer tissues and corresponding normal tissues. (b) The protein expression of STC2 in the specimens from pancreatic cancer patients. Immunohistochemistry staining with anti-STC2 antibody in tumor and surrounding normal tissues. (c) The patients were divided into two groups according to the tumor size, and the mRNA expression of STC2 was assessed in the two groups. (d) The mRNA expression of STC2 was analyzed in pancreatic cancer patients with or without lymph node metastasis. (e) The patients were divided into two groups based on the expression levels of STC2, and the five-year survival rate was determined during follow-up. (d) The AUC curve was used to evaluate the value of STC2 as a diagnosis biomarker for pancreatic carcinoma. *∗∗∗P*<0.001 was defined as very significant difference.

A total of 98 cases of clinical tissue samples were collected during surgery. The mRNA expression level of STC2 in pancreatic tumors was significantly elevated as compared to the surrounding normal tissues (Figure 1(a)). Consistently, the result of IHC showed that the protein expression of STC2 was remarkably upregulated in tumors as compared to the normal tissues (Figure 1(b)), to further study the significance of STC2 in the development of pancreatic cancer. The correlation between the expression of STC2 and the clinical parameters was examined. And the results showed that the elevated STC2 expression was closely associated with the tumor size (Figure 1(c)) and lymph node metastasis (Figure 1(d)).

For analysis of the expression of STC2 in 98 pancreatic cancer patients, the patients were divided into the high expression group and the low expression group, and the lower confidence limit of 95% CI (confidence interval) of median was set as a cut-off value, and the relative expression of STC2 was 3.51. The results showed that STC2 expression was significantly correlated with histology analysis (*p*<0.01), tumor size (*p*<0.05), and lymph node metastasis (*p*<0.05), which was consistent with the results of Figure 1 (Table 1). Given the close correlation between STC2 expression and clinicopathological factors, the prognosis of pancreatic cancer patients was analyzed by survival curve analysis. As shown in Figure 1(e), pancreatic cancer patients with high expression of STC2 represented a lower 5-year overall survival rate than the patients with low expression of STC2. And the Cox regression analysis with clinicopathological factors showed that the high mRNA expression of STC2 led to poor prognosis in pancreatic cancer patients (hazard ratio, HR=3.26, 95% CI: 2.34-8.64,* p*<0.01), which was slightly better than the predictive value of tumor size and lymph node metastasis (Table 2). Furthermore, in the receiver operating characteristic curve (ROC) analysis displayed in Figure 1(f), the area under curve (AUC) value was 0.8078 (*p*<0.0001).

### 3.2. Overexpression of STC2 Promoted the Proliferation of Pancreatic Cancer Cells

**Figure 2:**
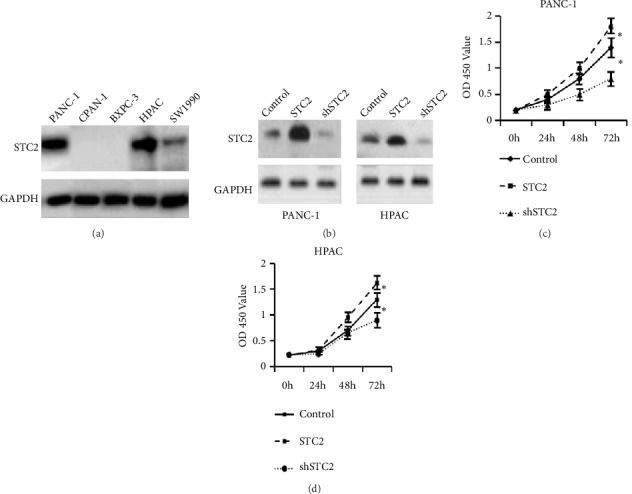
STC2 was involved in the regulation of proliferation of pancreatic cancer cells. (a) The expression of STC2 was detected in several pancreatic cancer cell lines. GAPDH was used as a loading control. (b) The efficiency of overexpression or knock-down of STC2 was assessed in PANC-1 and HPAC cells. (c) CCK-8 assay was subjected to evaluate the proliferation rate with overexpression or knock-down of STC2 in PANC-1 (left panel in (b)) or HPAC cells (right panel in (b)). The respective cells without treatment were confined as control cells. *∗P*<0.05 was considered as significant difference, and one-way analysis of variance (ANOVA) was used to analyze the difference. The results were represented as the mean ± standard deviation (n = 3).

To determine the function of STC2 in the pancreatic cancer cells, the expression of STC2 was assessed in five pancreatic cancer cell lines. As shown in Figure 2(a), the Capan-1 and BXPC-3 showed no expression, and PANC-1 and HPAC cell lines showed a relatively higher expression than SW1990 cells. To test the role of STC2 in the regulation of pancreatic cancer cells proliferation, the overexpression and knocking down constructs were transfected into PANC-1 and HPAC cells, and the successful construction of overexpression and knocking down of STC2 were characterized by western blotting (Figure 2(b)). After confirming the efficiency of overexpression and knock-down of STC2 in PANC-1 and HPAC cells, we further investigated whether STC2 could regulate their proliferation. As shown in Figure 2(c), overexpression of STC2 could promote the growth of PANC-1 and HPAC cells after transfection for 48 h. Conversely, knocking down STC2 could inhibit the proliferation of pancreatic cells.

### 3.3. Regulatory Role of STC2 in the Invasion and Migration of Pancreatic Cancer Cells

**Figure 3:**
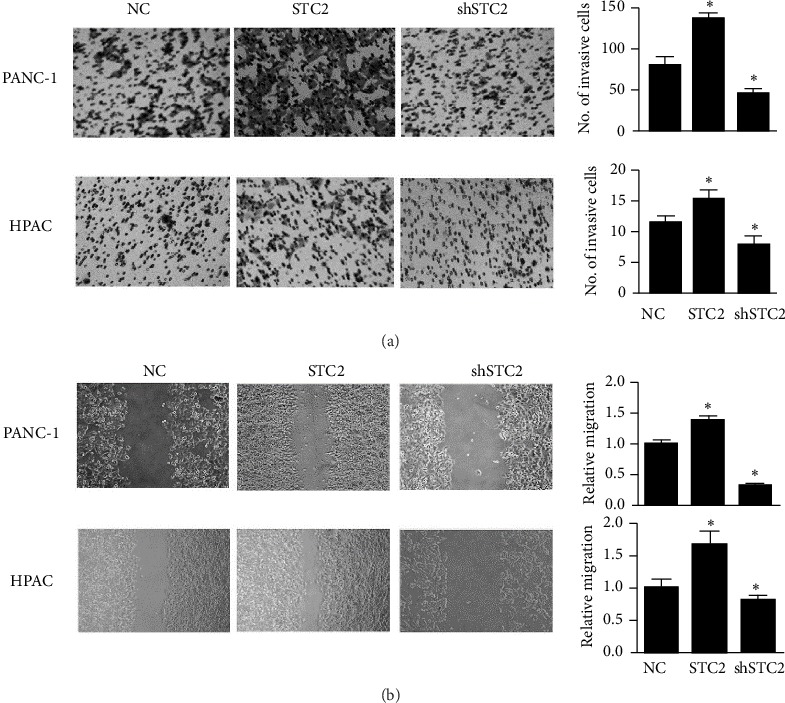
The effect of STC2 in migration and invasion of pancreatic cancer cells. (a) The transwell assay was used to test the effect of STC2 overexpression or knock-down in PANC-1 and HPAC cells. The right panel represents the mean cells in three fields of view. (b) The migration activity was assessed by the wound healing assay. The right panel shows the relative migration rate. The relative migration rate was quantified and compared with that of control cells. The respective cells with treatment were subjected as control cells. *∗* P<0.05 was significant difference between control cells. The results were represented as the mean ± standard deviation (n = 3).

Metastasis is an important cause of high mortality in pancreatic cancer. To determine the effect of STC2 on the metastasis of pancreatic cancer cells, the invasion and migration activities of PANC-1 and HPAC cells were assessed. The results showed that overexpression of STC2 could increase the invasion rate, while knocking down of STC2 decreased the invasion activity of pancreatic cancer cells (Figure 3(a)). Consistently, the wound healing assay showed that upregulation of STC2 elevated the migration activity of pancreatic cancer cells, while inhibition of STC2 downregulated the migration rate (Figure 3(b)).

### 3.4. STC2 Induced an Epithelial-to-Mesenchymal Transition in Pancreatic Cancer Cells

**Figure 4:**
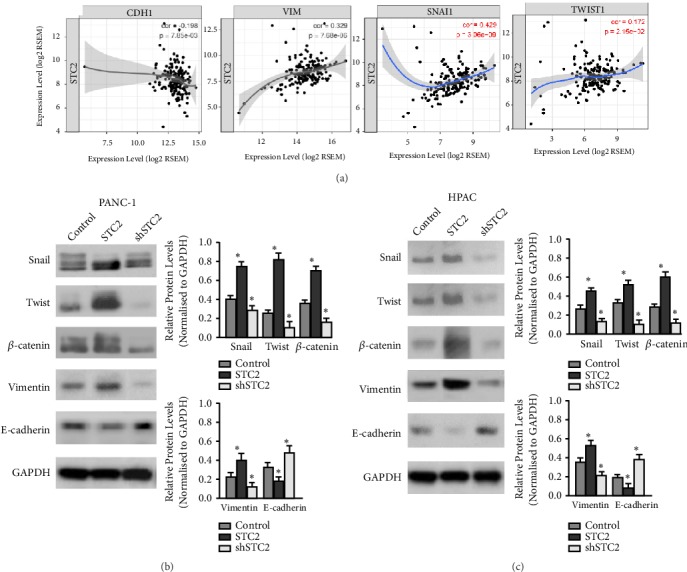
STC2 promoted the EMT process in pancreatic cancer cells. (a) The correlation curve between STC2 and E-cadherin (CDH1, left panel) and Vimentin (VIM, right panel) in TCGA pancreatic carcinoma database. (b) Overexpression or knock-down of STC2 in PANC-1 and (c) HPAC cells. Western blotting was subjected to analyze the expression of Snail, Twist1, *β*-catenin, Vimentin, and E-cadherin. GAPDH was used as a loading control. The relative expression levels were normalized to GAPDH. *∗p*<0.05, compared with vehicle controls. The results were represented as the mean ± standard deviation (n = 3).

The correlation between STC2 expression and the expression of epithelial markers such as E-cadherin (CDH1) or the mesenchymal marker such as Vimentin (VIM) were evaluated in 178 pancreatic cancer cases from the TCGA database, and the data revealed that the mRNA level of STC2 was negatively correlated with CDH1, and highly positively correlated with VIM. Moreover, STC2 was positively correlated with EMT-related molecules, snail1, and twist1; these data suggest a potential role of STC2 in EMT process (Figure 4(a)). To confirm the results, the expression of CDH1, VIM, and EMT related molecules such as Snail, Twist, and *β*-catenin were analyzed by western blotting in PANC-1 and HPAC cells. The results revealed that overexpression of STC2 could significantly reduce the expression of E-cadherin but increase the expression of Snail, Twist, *β*-catenin, and Vimentin expression (Figure 4(b)). In addition, silencing STC2 could remarkably increase E-cadherin expression and reduce the Snail, Twist, *β*-catenin, and Vimentin expression (Figure 4).

## 4. Discussion

STC2 appears to be a robust phosphoprotein implicated in the processes of tumor development and progression in several malignancies [10–13]. Furthermore, it serves as a promising marker for assessing the disease severity, and prediction of metastasis and prognosis [19]. We first determined the expression level of STC2 in pancreatic cancer, and the results showed that its expression had a good correlation with clinicopathological parameters, suggesting that STC2 was a potential biomarker for the diagnosis of pancreatic cancers. This was consistent with some previous studies. Law et al. showed that the STC2 expression was significantly correlated with tumor grade and histological type in ovarian cancer [8].

As shown by* in vitro* study, STC2 was involved in the regulation of pancreatic cancer proliferation. Similar roles were found in a few cancers. Wang et al. reported that STC2 promotes cell proliferation and cisplatin resistance in cervical cancer [9]. Yokobori et al. also showed that knocking down STC2 in gastsric cancer cell line reduced the cell proliferation [27].

Further results showed that STC2 modulated the invasion and migration of pancreatic cancer cells, suggesting a robust role of STC2 in this cancer. Similar results were also reported in liver and lung cancers. Wang et al. showed that STC2 was upregulated in hepatocellular carcinoma (HCC) and correlated with the tumor size and multiplicity of HCC [12]. The aberrant expression of STC2 promoted cancer cell growth, invasion, and colony formation while silencing STC2 delayed the cell cycle in G0/G1 phase. Further study revealed that STC2 regulated cyclin D1 and activated ERK 1/2. The overexpression of STC2 was observed in lung cancer cells, and knock-down of STC2 suppressed growth, colony formation, invasion, and metastasis of cancer cells [15]. The overexpression of STC2 in lung cancer tissues was also observed. Additionally, STC2 exerted a protective effect on the redox system of lung cancer.

EMT is an early event in the metastasis of cancer, which was widely reported as an important process for cancer development [28]. Given the evidence linking STC2 to tumor metastasis, the involvement of STC2 in the process of EMT was determined and we demonstrated that STC2 markedly induced an EMT transition in pancreatic cancer cells. This phenomenon was observed in colorectal cancer. Chen's study revealed that STC2 promoted EMT and colorectal cancer migration. Interestingly, the conditioned medium from EMT cells stimulated the epithelial cells to develop characteristics similar to those of EMT [11].

## 5. Conclusions

This study showed that overexpression of STC2 in pancreatic cancer contributed to the metastasis by promoting EMT, suggesting that STC2 could be a potential prognostic biomarker and therapeutic target for pancreatic cancer.

## Figures and Tables

**Table 1:** STC2 gene expression and clinicopathological factors in 98 pancreatic cancer patients.

Characteristics	STC2 expression		*p*-value
	High*∗* (n=60)	Low*∗* (n=38)	
Age (years, mean ± SD)	58.2 ± 5.7	60.3 ± 7.9	0.47
Sex (M/F)	38/22	20/18	0.29
Histology			
Well	8	14	0.006*∗*
Moderate/poor	52	24	
Tumor size			
	5	10	0.016*∗*
≥3cm	55	28	
Lymph node metastasis			
Yes	38	15	0.02*∗*
No	22	23	

*∗* The pancreatic carcinoma patients were divided into two groups based on the STC2 expression, and above the median level was identified as high expression groups and below the median level was classified as low expression group.

**Table 2:** Results of multivariate analyses of clinicopathological factors affecting overall survival rate after surgery.

Clinicopathological variable	Multivariate analysis	
	HR (95% CI)	*p* value
Gender (male/female)	1.35(0.84-2.05)	0.29
Histology (well/moderate, poor)	1.13(0.95-1.46)	0.08
Tumor size (<3cm/≥3cm)	2.43(1.15-4.63)	0.02*∗*
Lymph node metastasis (Yes/No)	2.84(1.33-5.75)	0.01*∗*
STC2 mRNA expression (low/high)	3.26(2.34-8.64)	0.006*∗*

## Data Availability

The data used to support the findings of this study may be released upon application to the Institutional Review Board of Fuzhou General Hospital (IR B00006161)
